# The Use of Lower or Higher Than Recommended Doses of Folic Acid Supplements during Pregnancy Is Associated with Child Attentional Dysfunction at 4–5 Years of Age in the INMA Project

**DOI:** 10.3390/nu13020327

**Published:** 2021-01-23

**Authors:** Laura María Compañ Gabucio, Manuela García de la Hera, Laura Torres Collado, Ana Fernández-Somoano, Adonina Tardón, Mònica Guxens, Martine Vrijheid, Marisa Rebagliato, Mario Murcia, Jesús Ibarluzea, Itxaso Martí, Jesús Vioque

**Affiliations:** 1 Instituto de Investigación Sanitaria y Biomédica de Alicante, ISABIAL-UMH, 03010 Alicante, Spain; lcompan@umh.es (L.M.C.G.); l.torres@umh.es (L.T.C.); vioque@umh.es (J.V.); 2 Unidad de Epidemiología de la Nutrición, Departamento de Salud Pública, Historia de la Ciencia y Ginecología, Universidad Miguel Hernández (UMH), 03550 Alicante, Spain; 3 CIBER Epidemiología y Salud Pública (CIBERESP), Instituto de Salud Carlos III, 28034 Madrid, Spain; fernandezsana@uniovi.es (A.F.-S.); atardon@uniovi.es (A.T.); monica.guxens@isglobal.org (M.G.); martine.vrijheid@isglobal.org (M.V.); rebaglia@med.uji.es (M.R.); murcia_mar@gva.es (M.M.); mambien3-san@euskadi.eus (J.I.); 4 IUOPA-Departamento de Medicina, Universidad de Oviedo, 33003 Oviedo, Spain; 5 Departamento de Epidemiología Ambiental y Molecular del Cáncer, Instituto de Investigación Sanitaria del Principado de Asturias (ISPA), Roma Avenue s/n, 33001 Oviedo, Asturias, Spain; 6 Department of Childhood & Environment, Barcelona Institute for Global Health-ISGlobal, 08036 Barcelona, Spain; 7 Departament de Ciències Experimentals i de la Salut, Universitat Pompeu Fabra (UPF), 08002 Barcelona, Spain; 8 Department of Child and Adolescent Psychiatry, Erasmus MC, University Medical Centre, 3015 GD Rotterdam, The Netherlands; 9 Epidemiology and Environmental Health Joint Research Unit, Foundation for the Promotion of Health and Biomedical Research in the Valencian Region, FISABIO-Public Health, FISABIO-Universitat Jaume I-Universitat de València, 46015 Valencia, Spain; 10 Department of Medicine, Universitat Jaume I, 12071 Castellón de la Plana, Spain; 11 Servicio de Análisis de Sistemas de Información Sanitaria, Conselleria de Sanitat, Generalitat Valenciana, 46010 Valencia, Spain; 12 Group of Environmental Epidemiology, Biodonostia Health Research Institute, 20014 Donostia-San Sebastián, Spain; itxasomarti@gmail.com; 13 Ministry of Health of the Basque Government, Sub-Directorate for Public Health and Addictions of Gipuzkoa, 20013 San Sebastián, Spain; 14 School of Psychology, University of the Basque Country UPV/EHU, 20018 San Sebastián, Spain; 15 Pediatric Neurology Department, Donostia University Hospital, Osakidetza Basque Health Service, 20014 San Sebastián, Spain

**Keywords:** folic acid, supplement use, pregnancy, attentional function, neurodevelopment, children

## Abstract

We assessed the association between the use of lower- and higher-than-recommended doses of folic acid supplements (FAs) during pregnancy and attentional function in boys and girls at age of 4–5. We analyzed data from 1329 mother-child pairs from the mother-child cohort INfancia y Medio Ambiente Project (INMA) study. Information on FAs use during pregnancy was collected in personal interviews at weeks 12 and 30, and categorized in <400, 400–999 (recommended dose), and ≥1000 μg/day. Child attentional function was assessed by Conners’ Kiddie Continuous Performance Test. Multivariable regression analyses were used to estimate incidence rate ratios (IRR) and beta coefficients with 95% confidence intervals (CI). Compared to recommended FAs doses, the periconceptional use of <400 and ≥1000 μg/day was associated with higher risk of omission errors—IRR = 1.14 (95% CI: 1.01; 1.29) and IRR = 1.16 (95% CI: 1.02; 1.33), respectively. The use of FAs < 400 μg/day and ≥1000 μg/day was significantly associated with deficits of attentional function only in boys. FAs use < 400 μg/day was associated with higher omission errors with IRR = 1.22 and increased hit reaction time (HRT) β = 34.36, and FAs use ≥ 1000 μg/day was associated with increased HRT β = 33.18 and HRT standard error β = 3.31. The periconceptional use of FAs below or above the recommended doses is associated with deficits of attentional function in children at age of 4–5, particularly in boys.

## 1. Introduction

An adequate dietary intake and periconceptional use of folic acid supplements during the embryonic period is crucial in several fetal physiological and cellular processes [1] that are essential for the optimal early and late development of the child’s brain and cognitive function [2,3]. The folic acid (synthetic form of folate) is an essential micronutrient required for many metabolic processes such as the metabolism of carbon-1 and for S-adenosylmethionine (SAM) production [4], nucleotide synthesis, DNA repair, methylation reactions, myelination, and synthesis of neurotransmitters [5,6]. There is also evidence from animal studies suggesting that the effect of folic acid supplements (FAs) modulating DNA methylation reactions and the brain development of the child, may be different according to sex [7].

Based on the initial results of the Medical Research Council (MRC) clinical trial and subsequent evidence, a daily dose of 400 μg/day from folic acid (FA) in the periconceptional period is recommended to reduce the risk of neural tube defects [8,9]. Thus, in many countries, women planning to become pregnant are advised to take this dosage of FAs, to avoid exceeding the tolerable upper limit of 1000 μg/day, and to eat varied diets rich in dietary folate. In Spain, there is no information on FA status at national level, although in a population-based mother-child cohort study in Valencia, the periconceptional mean daily FA intake was 304 μg, lower than the recommended dietary intake [10]. The proportion of women using FA supplements in preconception, the first month, and the second month of pregnancy was 19%, 30%, and 66% respectively; and among women using FA supplements in the periconceptional period, 30% exceeded the upper tolerable limit (UTL) for FAs of 1.000 μg/day [10].

Recently published studies suggest that the use of FAs during pregnancy at doses below or above the present recommendations during the periconceptional period of pregnancy may cause changes in the fetal brain, which can alter the future child’s cognitive and motor processes [11,12]. In fact, a deficient FAs use in pregnancy has been associated with poorer cognitive skills in children [13], and in our study over a half of pregnant women used low doses of FAs (<400 μg/day, including no use) [14]. Moreover, one-third of the pregnant women in our study used high FAs doses above 1000 μg/day (3.5% consuming >5000 μg/day), which has been also related to psychomotor delay at one year of age [15], and cognitive deficits at 4–5 years of age [16], especially in verbal function. In this sense, early childhood and preschool age are appropriate periods to investigate the potential cognitive effects of the use of FAs, since the prefrontal cortex, the brain area related to superior cognitive functions, achieves its synaptic formation peak at the age of 4 years, approximately [17,18].

The negative effects of low or high of FAs doses on cognitive function have been also shown in an animal studies with mice models. In one study, three type of diets were used: one group containing no FA (low doses), another group with high doses of FA (20 mg/kg), and a control group with the recommended doses (2 mg/kg). Compared to offspring from the control group, those from the low and high doses groups presented structural and physiological neurodevelopmental abnormalities such as thinner cortex, persistent deviation in cortical cytoarchitectural organization, less complex dendritic arbors (which could be related to neuronal dysfunction [19], delay in prenatal neurogenesis, and an increased apoptosis in the developing cortex. In addition, compared to mice exposed to recommended doses, the FA-deficient offspring underperformed in memory tests and the exposed to high FA doses showed an increased open space-induced anxiety [20].

Nevertheless, little is still known regarding the effect of high or low FAs doses in children’s attentional function. Attentional function is crucial to children’s social relations and learning [21]. It is a superior cognitive function, which involves different processes, such as focus on a specific stimulus during a period of time to process it adequately, while ignoring other stimuli from the environment [22]. Their development begins in the embryonic period, in which the brain is very vulnerable to environmental factors such as the mother’s diet [23,24], and it continues until adolescence [25,26]. A recently published review [27] describes the importance of the maternal use of FAs at recommended doses in order to reduce inattention symptoms in school-age children and improve attention and concentration in adolescents. Thus, our hypothesis is that the use of lower (<400 μg/day) or higher (≥1000 μg/day) doses of FAs during pregnancy compared to the recommended use (400–999 μg/day) may produce attentional dysfunction in children at 4–5 years of age. Therefore, we aim to evaluate the association between the use of FAs at doses below or above the recommended in pregnancy and attentional function in children at 4–5 years of age.

## 2. Materials and Methods

### 2.1. Study Design and Population

**Figure 1:**
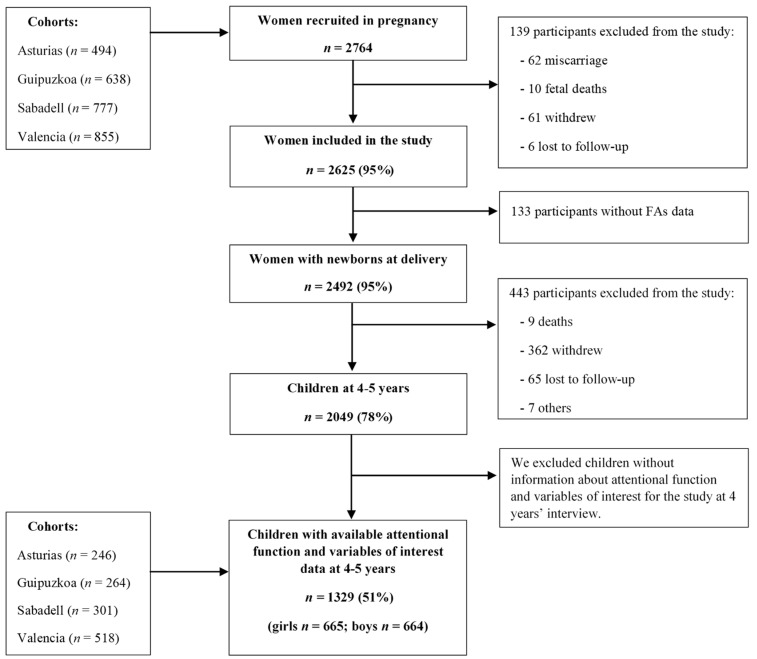
Flowchart of the study population describing the selection process.

The INMA—INfancia y Medio Ambiente [Environment and Childhood] Project—is a population-based prospective birth cohort study, established in seven regions of Spain following a common protocol [28]. In the current study, we used the data collected from the INMA regions of Valencia, Sabadell (Catalonia), Asturias, and Gipuzkoa (Basque-country). These four cohorts were established between 2003 and 2008. Detailed information on the population and study design has been published elsewhere [28]. Briefly, participants were recruited during the first prenatal visit, between weeks 10 and 13 of gestation at public primary health care centers or public hospitals. Women who agreed to participate were followed up at the third trimester of gestation; delivery; when children were 1 year of age; and at 4–5 years of age. Of the available 2764 women, 139 women were excluded from the study due to withdrawal, being unavailable for follow-up, induced or spontaneous abortions, or fetal deaths. A total of 2625 women delivered a live infant between May 2004 and August 2008, and 2049 children participated at the 4-year follow-up assessment. Finally, for the present study, 1329 mother-child pairs were included with available data on the main variables when children were aged 4 to 5. Figure 1 shows the flowchart of the population sample in our study. The final analysis was based on 1329 children since the attentional function assessment was introduced late in the 4–5 years follow-up. All participants gave informed written consent in each phase of the study prior to participation, and the Institutional Ethical Committees of the participating centres involved in the study approved the research protocol (CEIC-Hospital La Fe, Valencia; CEIC-Hospital de Zumárraga, Gipuzkoa; CEIC-Parc de Salut Mar, Barcelona; CEIC-Hospital Universitario Central de Asturias).

### 2.2. Dietary Folate and FAs Intake during Pregnancy

The assessment of dietary folate and FAs intake has been described previously [15,29] (available at: http://epinut.edu.umh.es/cfa-101-inma-embarazadas/). Briefly, validated food frequency questionnaires (FFQs) [30] were administered to pregnant women at 10–13 and 28–32 weeks to estimate dietary intakes from three months preconception to the third month of pregnancy, and from the fourth to the seventh month of pregnancy, respectively. The folate content of food was primarily obtained from USDA food composition tables [31] and other Spanish-published sources [32]. In the present study, we estimated the typical dietary folate intake for the periconceptional period, for the second–third trimester of pregnancy, and for the average of these two periods combined (entire pregnancy).

The consumption of FAs or vitamin and mineral preparations containing FAs were obtained through specific supplement questions of the FFQ. FAs use was estimated based on daily dose, supplement brand name and composition, and duration of consumption for each period of pregnancy. We estimated the monthly use of FAs and the average for each woman in periconceptional period, the second period of pregnancy, and during the entire pregnancy. After that, we categorized the use of FAs in these periods as <400 µg/day, 400–999 µg/day (this was used as the reference category in the analyses), and ≥1000 µg/day, similarly as we have done in previous work [16].

### 2.3. Children’s Attentional Function Assessment

Attentional function was measured at the median age of 4.6 years using Conner’s Kiddie Continuous Performance Test (K-CPT) [33] although in Valencia, the median age of children was 5.8 years. The K-CPT (K-CPT TM v.5) is a 7.5-min computerized test that examines inattention, impulsivity, sustained attention, and vigilance in children aged 4 to 7 years [34]. This test was administered to children individually in a quiet room by trained fieldworkers. Children were instructed to press the space bar on the keyboard as fast as possible when they saw an image on the computer screen (the target), unless that image was a ball (the non-target). The main five interest measures of the K-CPT included (Table S1): omission errors (i.e., the number of times the child did not respond to a target); commission errors (i.e., the number of times the child responded erroneously to a non-target); hit reaction time (HRT, the mean response time—expressed in milliseconds—for all correct responses during the entire test); standard error of the hit reaction time [HRT(SE); the within-child variability of the HRTs, which is a measure of speed response consistency—expressed in milliseconds—throughout the test [35], and finally, the detectability of attentiveness (d’, a measure—without units—which shows the children’s capacity to distinguish a target from a non-target stimulus [33,36]). Higher scores in the K-CPT outcomes mean adverse attention results, with the exception of detectability.

### 2.4. Other Variables

Information on potential confounders were obtained from questionnaires completed at the personal interview in pregnancy. These questionnaires included information on sociodemographic and lifestyle factors such as: mother’s characteristics (location (Asturias; Gipuzkoa; Sabadell; Valencia), age (in years), energy intake (in kilocalories), social class (I + II [high], III [medium], or IV + V [low]), educational level (primary or less, secondary, or university), parity (0 or ≥1), overall tobacco exposition (yes/no), BMI pre-pregnancy (continuous)); father’s BMI (continuous); and child’s characteristics (sex (male, female) and age at K-CPT examination (in years)).

### 2.5. Statistical Analysis

Descriptive analysis of sociodemographic, lifestyle, and obstetric characteristics of pregnant women was performed for the four areas of the INMA project. To explore the normal distribution of the quantitative variables, we used the Kolmogorov test. Moreover, we used the Chi-square test for qualitative variables and the Kruskal-Wallis test for quantitative variables.

All the covariates with *p* value >0.20 in bivariate analysis and those that changed the magnitude of the main effects by >10% after a backward elimination procedure were included in the core model. Multiple robust linear regression models using an MM-type estimator was performed to evaluate the association between FAs use and K-CPT outcomes (HRT, HRT (SE), and detectability) for each pregnancy period: the periconceptional period, second period of pregnancy, and throughout the entire pregnancy [37]. Multiple negative binomial regression models were used to estimate associations between FAs use and K-CPT count outcomes (number of omission errors and number of commission errors) for each period of pregnancy [38]. The regressions coefficients of negative binomial models were exponentiation to obtain IRR (incidence rate ratios) which should be interpreted as relative risk. Besides this, to obtain combined estimates for the four study cohorts, we used a meta-analysis. Heterogeneity was quantified with the I2 statistic under the fixed-effects hypothesis (I2 ≤ 50%), and when heterogeneity was detected (I2 > 50%), the random effects model was used [39]. To verify potential effect modification, additional models stratified by sex of the children were run.

Sensitivity analysis was performed to analyze the robustness of the main findings. We added mother or child conditions that could be related to child cognitive function development as a different adjustment to the basal model. So, we included in the basal model maternal variables such as iodine intake from supplements; fish consumption during pregnancy; verbal reasoning outcome of the similarities subtest of the Wechsler Adult Intelligence Scale-III; time of initiating FAs usage; NO_2_ exposition during pregnancy; and use of acetaminophen during pregnancy. In addition, we ran the basal model with child variables such as dietary folate intake assessed by validated FFQ. Finally, to avoid any potential influence in the association, we conducted the basal model after excluding subjects with some conditions such as preterm deliveries (*n* = 1273), mothers with medical conditions that could affect the typical development of cognitive functions (diabetes mellitus, epilepsy, or thyroid disease) (*n* = 1079), mothers who did not use FAs throughout their entire pregnancies (*n* = 1258), children who met the WHO obesity criteria (97th percentile) (*n* = 1192), children with psychomotor delay on the Bayley Scales of Infant and Toddler development (BSID) at age 1 year (<85th percentile) (*n* = 1207), and children with low scores in executive function on the McCarthy Scales of Children’s Abilities (MSCA) (<76.3 score) (*n* = 1248). We also performed sensitivity analyses stratifying by boys and girls.

Statistical analyses were conducted with R 3.4.2 (R Foundation for Statistical Computing).

## 3. Results

The baseline characteristics of the pregnant women and their children according to the four areas of the study are shown in Table 1. Overall, the median age of the mothers was 31 years, more than a third (36.3%) had completed university studies, and almost half of them (48.8%) had low social class.

A total of 55.8% of the mothers used <400 µg/day FAs doses and 29.2% used ≥1000 µg/day FAs doses in the periconceptional period. In this period, the highest proportion of pregnant women taking <400 µg/day FAs doses were from Sabadell (63.1%), while the highest proportion of pregnant women taking ≥1000 µg/day FAs doses were from Asturias (37.4%) (χ2 = 26.986, 6 d.f., *p* = 0.00014). In the second period of pregnancy, 51.4 and 18.1% of the mothers used doses of <400 µg/day and ≥1000 µg/day FAs, respectively. In this period, an important proportion of mothers from Valencia (19.9%) and Asturias (37.4%) continued taking ≥1000 µg/day FAs doses (χ2 = 350.2, 6 d.f., *p* < 0.0000002) (Table 1). Children from Valencia showed lower results in most KCP-T outcomes compared to children from other study areas, although they showed the highest scores in the number of commission errors together with children from the Sabadell area (Kruskal-Wallis χ2 = 24.823, 3 d.f., *p* = 0.000016) (Table 1).

**Figure 2:**
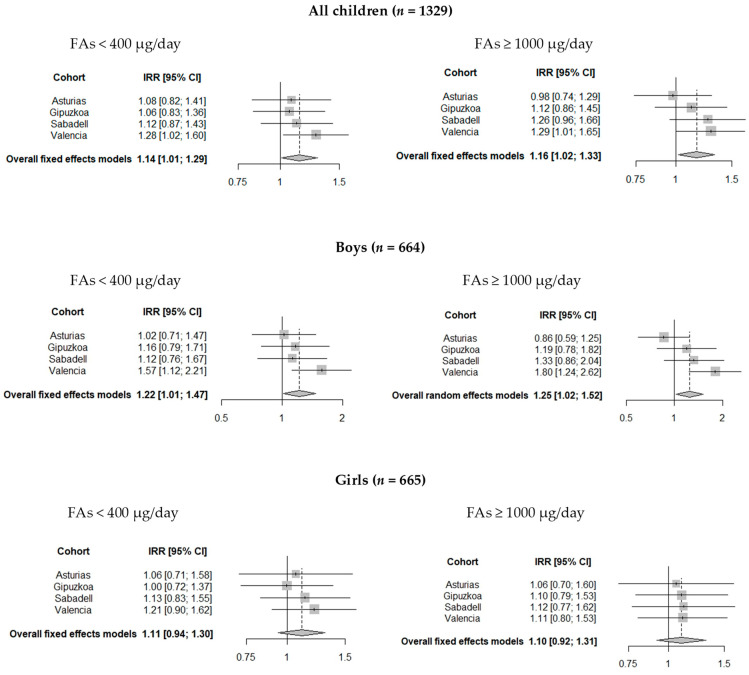
Pooled estimates of the associations between FAs (<400 and ≥1000 µg/day compared to 400–999 µg/day) and number of omission errors during periconceptional period of pregnancy in all children, boys, and girls at 4–5 years of age.

Table 2 presents the fully-adjusted models with the combined associations between use of FAs and K-CPT outcomes in the different periods of pregnancy. Compared to the periconceptional use of recommended FAs doses (400–999 µg/day), the periconceptional FAs use of <400 µg/day and ≥1000 µg/day was associated with a 14% (IRR = 1.14; 95% CI = 1.01 to 1.29) and 16% (IRR = 1.16; 95% CI = 1.02 to 1.33) of increase in the number of children’s omission errors, respectively. The associations between the periconceptional use of non-recommended FAs doses and the number of omission errors in each area of study were similar to the combined results, but in the Valencia area the number of associations was slightly higher and reached statistical significance (Figure 2). There were no statistically significant associations in other K-CPT outcomes or in other periods of pregnancy. We explored the interactions between overall tobacco exposition and the use of FAs during the periconceptional period but none were significant.

**Figure 3:**
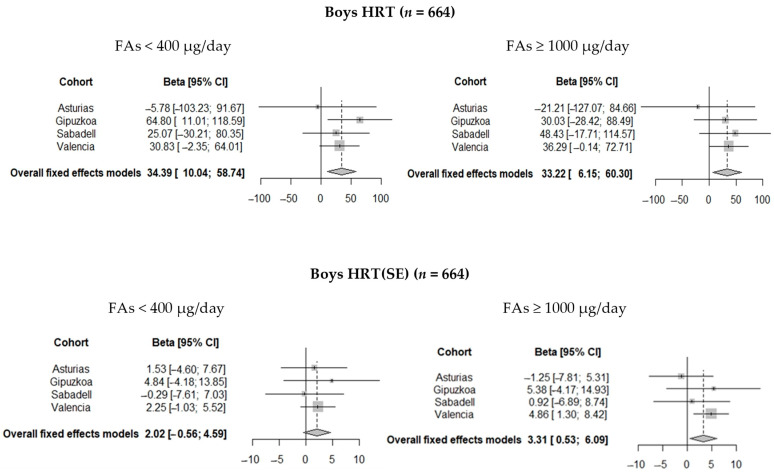
Pooled estimates of the associations between FAs (<400 and ≥1000 µg/day compared to 400–999 µg/day) and Conner’s Kiddie Continuous Performance Test (K-CPT) outcomes in boys at 4–5 years of age.

When the analysis was stratified by sex, the positive association found between periconceptional FAs use of <400 µg/d and the increased number of omission errors was only observed in boys (IRR = 1.22; 95% CI = 1.01 to 1.47), this FAs use was also associated with a higher HRT in boys (β = 34.36; 95% CI = 10.01 to 58.71) (Figure 2). In addition, the periconceptional FAs use of ≥1000 µg/day was associated with a higher HRT in boys (β = 33.18; 95% CI = 6.10 to 60.25) and a higher HRT (SE) (β = 3.31; 95% CI = 0.53 to 6.09). In girls, we only observed a protective association between the FAs use of <400 µg/day during the second period of pregnancy and decreased number of omission errors (IRR = 0.86; 95% CI = 0.74 to 1.00) (Table 3). The combined associations between non-recommended FAs doses and boys’ and girls’ attentional function outcomes were similar to those observed by cohort. Omission errors and HRT(SE) associations found in boys were slightly higher and reached statistical significance only in the Valencia area, whereas the HRT association found in boys reached statistical significance only in the Gipuzkoa area (Figure 3).

**Figure 4:**
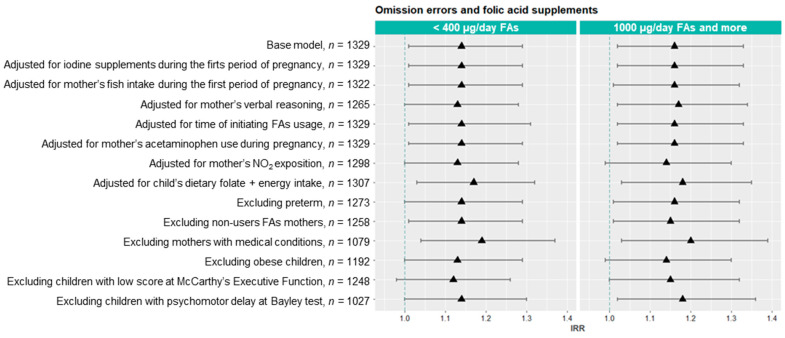
Sensitivity analyses of the associations between FAs use (<400 and ≥1000 µg/day compared to 400–999 µg/day) in the periconceptional period and number of omission errors in all children (*n* = 1329), girls (*n* = 665) and boys (*n* = 664) at 4–5 years.

Sensitivity analyses were conducted to assess the robustness of the association between the periconceptional use of non-recommended FAs doses and the number of omission errors under a variety of scenarios in all children and by sex (Figure 4). In all children, the associations between FAs use of <400 µg/day and the number of omission errors remained similar to the basal model (IRR = 1.14; 95% CI = 1.01 to 1.29), although we observed a stronger association when we adjusted for child dietary folate and energy intake (IRR = 1.17; 95% CI = 1.03 to 1.32) and when we excluded mothers with medical conditions (IRR = 1.19; 95% CI = 1.04 to 1.37). In addition, the association between the FAs use of ≥1000 µg/day and omission errors in the basal model (IRR = 1.16; 95% CI = 1.02 to 1.33) became slightly stronger when we excluded those mothers with medical conditions (IRR = 1.20; 95% CI = 1.03 to 1.39). As with the all-children associations, associations between FAs use of <400 µg/day and the number of omission errors in boys remained very similar to the basal model (IRR = 1.22; 95% CI = 1.01 to 1.47), although it became stronger when we adjusted for mother’s acetaminophen use during pregnancy (IRR = 1.24; 95% CI = 1.03 to 1.49) and when we excluded mothers with medical conditions (IRR = 1.26; 95% CI = 1.02 to 1.56). Finally, when we added other variables such as alcohol consumption in grams day or breastfeeding (no, <20 weeks, or ≥20 weeks) in multivariable models, the observed associations remained similar and still significant. Similarly, after excluding children with missing values or scoring less than 7 in the Apgar test at 1 or 5 min after birth, the results were unchanged (data not shown).

## 4. Discussion

In the present study, we found that, compared to the periconceptional use of the recommended FAs doses, the use of FAs doses <400 or ≥1000 µg/day was associated with an increase in the number of omission errors in children at 4–5 years of age. Moreover, our results suggest that the association may be sex-specific, since only boys presented an increase in the number of omission errors with mothers taking <400 µg/day of FAs, and an increase of speed response consistency throughout the test (HRT(SE)) in mothers taking ≥1000 µg/day.

In our study, 266 out of 1329 children scored above the 80th percentile in the number of omission errors in the K-CPT assessment at the age of 5 years, which may be indicative of inattention. A prevalence of 18% of inattention in children of 5 years of age was found in a birth cohort study from United States [40]. In two birth cohorts in China, prevalences of 6.4% [41] and 12.3% [42] were reported for children 3 and 4 years old, respectively, although they evaluated the prevalence of Attention-Deficit-Hyperactivity Disorders (ADHD) while we evaluated only attentional function—just one of the ADHD symptoms. In another birth cohort study from Denmark, a prevalence of 30% was reported for children of 5 years [43]. Thus, it is difficult to make comparisons of inattention prevalence among studies since specific cut-off points or definitions for inattention are not used consistently yet.

To our knowledge, this is the first prospective study that suggests an increase in the number of omission errors in children aged 4–5 years with mothers taking non-recommended FAs doses (<400 or ≥1000 µg/day) compared to mothers taking recommended FAs doses (400–999 µg/day) during the periconceptional period of pregnancy.

On the one hand, we found a negative effect of the periconceptional FAs use of <400 µg/day on children’s attentional function at 4–5 years of age, specifically an increased number of omission errors, which is an indicative of inattentiveness [44]. This is a relevant finding that highlights the need to monitor the compliance of using the recommended FAs doses among pregnant women to avoid negative effects on the cognitive function of their children. The evidence evaluating the use of non-recommended FAs doses during pregnancy is very scarce due to ethical and moral commitments. Therefore, hypotheses like ours can only be evaluated in prospective birth cohort studies such as the INMA Project. In one birth cohort study with 420 children in Menorca, Julvez et al. [45] also showed a significant lower risk of inattention symptom at 4–5 years of age among women using periconceptional FAs. Similarly, del Río García et al. have shown that maternal dietary folate deficiency has been related to lower BSID mental scores in 253 Mexican infants [46]. This detrimental effect on attentional function could be related to the maternal folate deficiency that could affect early brain development [47], and result in a poorer offspring cognitive function [13]. In fact, a recently published study has shown higher apoptosis level in brain cells of mice offspring fed with a deficient FA diet [39], which could also affect offspring cognitive function.

On the other hand, we found a negative effect of the periconceptional FAs use of ≥1000 µg/day on children’s attentional function at 4–5 years of age. In a similar way, negative effects on other domains of neurodevelopment have been reported in previous studies of the INMA project. Thus, an association between high periconceptional doses of FAs and negative effects on the development of offspring [15,16] has been reported, especially with negative effects on the cognitive development at age of 4–5 years [16]. These results have also been confirmed in animal studies. A growing number of animal researches have displayed that high doses of FAs has been associated with the presence of plasma unmetabolized FA producing alterations in the methylation pattern of several key developmental genes in offspring [48] and alterations in the expression of genes in the frontal cortex of offspring pups [49]. Considering that the frontal cortex is one of the most important parts of the brain related to attentional function, we could contemplate this as an explanation of the negative influence of high doses of FAs during periconceptional period on attentional function of children at 4–5 years of age. All of this evidence, including our results, offer safety concerns on the potential harmful effects of the use of high doses of FAs throughout the whole life as described by Selhub and Rosenberg [50], particularly for children’s cognitive function.

Moreover, we found sex differences in some attentional function outcomes. Boys of mothers using periconceptional FAs of <400 µg/day had increased omission errors and HRT, and boys of mothers using periconceptional FAs of ≥1000 µg/day had an increased speed response consistency throughout the test [HRT(SE)] and HRT compared to mothers using periconceptional recommended FAs doses. The associations found in girls are also negatives, but none reached statistical significance. Similarly, a recent study of INMA Project found sex differences on neuropsychological development at 4–5 years of age, being boys more vulnerable to prenatal pollutants exposure [51]. These results may indicate that boys are more vulnerable to a deficient periconceptional FAs use than girls. We did not find other studies that explore the effects of FAs use during pregnancy on neuropsychological development depending on the children’s gender. However, a similar result in the KCP-T test was found in children participating in the Health Outcomes and Measures of the Environment (HOME) Study [52] in which omission errors were associated with executive difficulties only in boys at 5 years of age. Brain model system studies using image technologies could help us to understand the neurological basis of this sex difference in attentional function outcomes. A recent study analyzed sex and age-related brain changes in children and showed several differences between boys’ and girls’ brain development, including a higher connectivity in brain areas related to attentional function, namely superior and left orbitofrontal cortex, in boys compared to girls [53]. Furthermore, animal studies can partly explain these differences by the fact that sexual distinctions in brain neurodevelopment emerge during gestation, including the functional connectivity of the prefrontal cortex. During gestation, girls demonstrated a longer range of changes in functional connectivity in prefrontal cortex compared to boys; furthermore, this connectivity decreased as gestational age increased more pronouncedly in boys [54]. Consequently, it is possible that boys need a greater contribution of FAs during earlier stages of gestation than girls because it is the moment in which they present a greater connectivity, and therefore are more sensitive to low or high periconceptional FAs doses.

Finally, we observed stronger associations in the Valencia area between K-CPT outcomes (omission errors and HRT(SE)) in all children as well as in both boys and girls when we performed pooled estimates. These differences could be in part explained by the fact that children from Valencia were older than the children of the other areas of study at the K-CPT examination. In this sense, studies using magnetic resonance imaging (MRI) technology have described the functional connections in the whole brains of healthy children and found that numerous global network parameters showed significant increases with age [53] and a progressive functional maturation of attention brain allocation in adolescents [55].

This study has several limitations. We adjusted for a wide range of potential risk factors, although we cannot discard potential residual confounding due to not including potential confounding variables in the analyses, or bias due to subjects lost to follow up. However, mothers of children who performed the K-CPT assessment, presented practically equal age, educational, and socioeconomic statuses and FAs use, compared to those mothers who were excluded due to unavailability of data for K-CPT test. Moreover, estimates from self-reported supplementation may cause some misclassification, but any inaccuracy in reporting should be non-differential. In this sense, to classify women according to their FAs use, we used a FFQ which may be limited in estimating exact absolute values of folate intake, but it showed an acceptable reproducibility for folate intake (*r* = 0.48) and biochemical validity (*r* = 0.53) [30]. Finally, our results do not provide a possible explanation of the mechanisms by which the use of non-recommended doses of FAs during pregnancy could produce attentional dysfunction at 4–5 years of age. However, they can constitute an argument to future research in this field.

The major strengths of this study include a large sample size and a prospective design that minimized recall and selection bias. Additionally, we used a validated FFQ test to estimate FAs intake and a standardized and computerized battery for K-CPT to evaluate attentional function, which may detect small changes on responses in children and provide objective assessment. Although not specifically validated, these tests were administrated by trainer interviewers which provided more unbiased assessment. Furthermore, the results from sensitivity analysis reinforced the consistency of these findings. Finally, the multicenter structure of the INMA Project showing different patterns of FAs use and the detailed information on the doses of FAs used at each period of pregnancy, including a crucial period of pregnancy, namely periconceptional period, may add value to our results.

## 5. Conclusions

In conclusion, this prospective mother–child cohort study shows that the periconceptional non-recommended FAs doses (<400 µg/day or ≥1000 µg/day) compared to the FAs use of 400–999 µg/day may have negative effects on attentional function development in children at 4–5 years of age. Furthermore, the use of non-recommended FAs doses could have different effects in girls and boys. Our findings add to existent evidence supporting a potential adverse effect of the use of non-recommended FAs doses, so we suggest not using these doses, unless medically prescribed. Nevertheless, additional studies are needed to confirm the associations found between non-recommended FAs doses and attentional function in children.

## Figures and Tables

**Table 1:** Socio-demographic, lifestyle, and obstetric characteristics of parents and children at four years, 2003.

	All Cohorts (*n* = 1329)	Valencia (*n* = 518)	Sabadell (*n* = 301)	Asturias (*n* = 246)	Gipuzkoa (*n* = 264)	*p* ^1^
Mother’s age, year	31 (28–34)	30 (28–33)	31 (28–34)	31 (29–35)	31 (29–33)	<0.001
Educational level						
Primary or less	285 (21.4)	144 (27.8)	73 (24.3)	40 (16.3)	28 (10.6)	<0.001
Secondary	561 (42.2)	226 (43.6)	121 (40.2)	111 (45.1)	103 (39.0)	
University	483 (36.3)	148 (28.6)	107 (35.5)	95 (38.6)	133 (50.4)	
Social class						
I + II (high)	312 (23.5)	99 (19.1)	70 (23.3)	60 (24.4)	83 (31.4)	<0.001
III	368 (27.7)	138 (26.6)	100 (33.2)	56 (22.8)	74 (28.0)	
IV + V (low)	649 (48.8)	281 (54.2)	131 (43.5)	130 (52.8)	107 (40.5)	
Parity ≥ 1	564 (42.4)	234 (45.2)	129 (42.9)	92 (37.4)	109 (41.3)	0.230
Overall tobacco exposition during pregnancy, yes	810 (61.0)	370 (71.4)	179 (59.5)	108 (43.9)	153 (58.0)	<0.001
Missing values	26 (1.3)	6 (1.2)	6 (2.0)	12 (4.9)	2 (0.8)	
Prepregnancy mother’s BMI, kg/m^2^						
<18.5–25	982 (73.9)	368 (71.0)	232 (77.1)	172 (69.9)	210 (79.5)	0.045
>25–30	251 (18.9)	103 (19.9)	50 (16.6)	58 (23.6)	40 (15.2)	
>30	96 (7.2)	47 (9.1)	19 (6.3)	16 (6.5)	14 (5.3)	
Prepregnancy father’s BMI, kg/m^2^						
<18.5–25	569 (42.8)	226 (43.6)	142 (47.2)	75 (30.5)	126 (47.7)	<0.001
>25–30	574 (43.2)	233 (45.0)	112 (37.2)	120 (48.8)	109 (41.3)	
>30	164 (12.5)	59 (11.4)	43 (14.3)	42 (17.1)	20 (7.6)	
Missing values	22 (1.7)	0 (0.0)	4 (1.3)	9 (3.7)	9 (3.4)	
Child’s sex, male	664 (50.0)	267 (51.5)	153 (50.8)	121 (49.2)	123 (46.6)	0.600
Child’s sex, female	665 (50.0)	251 (48.5)	148 (49.2)	125 (50.8)	141 (53.4)	
Age at K-CPT examination, year	4.6 (4.4–5.7)	5.8 (5.7–5.8)	4.5 (4.4–4.6)	4.4 (4.3–4.5)	4.5 (4.4–4.5)	<0.001
Dietary folate, µg/day						
First period	294 (237–359)	294 (234–360)	282 (231–343)	312 (245–370)	307 (246–365)	0.003
Second period	291 (235–358)	278 (220–353)	289 (233–348)	300 (242–365)	302 (259–369)	<0.001
FAs µg/day						
First period						
<400	742 (55.8)	309 (59.7)	190 (63.1)	112 (45.5)	131 (49.6)	<0.001
400–999	199 (15.0)	80 (15.4)	35 (11.6)	42 (17.1)	42 (15.9)	
≥1000	388 (29.2)	129 (24.9)	76 (25.2)	92 (37.4)	91 (34.5)	
Second period						
<400	683 (51.4)	169 (32.6)	252 (83.7)	66 (26.8)	196 (74.2)	<0.001
400–999	405 (30.5)	246 (47.5)	35 (11.6)	88 (35.8)	36 (13.6)	
≥1000	241 (18.1)	103 (19.9)	14 (4.7)	92 (37.4)	32 (12.1)	
K-CPT outcomes						
HRT, ms	705 (633–792)	669 (604–735)	718 (647–803)	758 (678–843)	733 (659–845)	<0.001
HRT(SE), ms	28 (20.6–37.9)	22.9 (17.2–30.7)	32.6 (24.7–43.6)	31.6 (24.1–40.0)	31.3 (22.9–41.6)	<0.001
Detectability, no unit	0.6 (0.3–0.9)	0.5 (0.3–0.8)	0.5 (0.3–0.8)	0.7 (0.3–1.0)	0.6 (0.3–0.9)	0.011
Omissions, number	18 (9–34)	10 (5–20.75)	24 (13–37)	23 (13–37.75)	31 (16.75–50)	<0.001
Commissions, number	21 (13–29)	22 (15–29)	22 (15–31)	16.5 (10–30.75)	18 (11–28)	<0.001

First period, periconceptional period of pregnancy; second period, from the fourth to the seventh month of pregnancy; FAs, folic acid supplement; HRT, hit reaction time (ms); HRT(SE), hit reaction time standard error (ms); INMA, Infancia y Medio Ambiente; K-CPT, Conners’ Kiddie Continuous Performance Test; ms, milliseconds; num, number. Values are medians (IQRs) for mother’s age, age at K-CPT examination, dietary folate, and K-CPT outcomes; and values are *n* (%) for the rest of variables. ^1^ *p* values of differences between study cohorts from the Chi-square test (categorical variables) and Kruskal-Wallis test (continuous nonparametric variables).

**Table 2:** Fully-adjusted combined association between folic acid supplement (FAs) use during pregnancy and attentional function outcomes in children aged 4–5 years of the Infancia y Medio Ambiente (INMA) cohort study, Spain, 2003–2008 (*n* = 1329).

	HRT			HRT(SE)			Detectability			Omissions			Commissions		
	β ^1^ (95% CI)	*p*	*I* ^2^	β ^1^ (95% CI)	*p*	*I* ^2^	β ^1^ (95% CI)	*p*	*I* ^2^	IRR ^1^ (95% CI)	*p*	*I* ^2^	IRR ^1^ (95% CI)	*p*	*I* ^2^
First period															
FAs, µg/day															
400–999	Ref.			Ref.			Ref.			Ref.			Ref.		
<400	14.56 (−3.26; 32.38)	0.109	0.0	1.01 (−0.69; 2.70)	0.244	0.0	0.03 (−0.11; 0.16)	0.685	75.5	1.14 (1.01; 1.29)	0.035	0.0	0.96 (0.83; 1.12)	0.633	66.1
≥1000	9.06 (−11.02; 29.14)	0.376	0.0	1.73 (−0.13; 3.60)	0.068	0.0	−0.00 (−0.16; 0.15)	0.986	79.0	1.16 (1.02; 1.33)	0.027	0.0	0.99 (0.83; 1.19)	0.941	72.3
Second period															
FAs, µg/day															
400–999	Ref.			Ref.			Ref.			Ref.			Ref.		
<400	3.80 (−11.23; 18.83)	0.620	0.0	−1.25 (−2.70; 0.21)	0.094	9.3	0.02 (−0.04; 0.08)	0.455	0.0	0.91 (0.82; 1.02)	0.097	0.0	0.97 (0.91; 1.04)	0.426	0.0
≥1000	−1.39 (−21.45; 18.67)	0.892	0.0	0.41 (−3.25; 4.07)	0.825	52.7	−0.02 (−0.09; 0.05)	0.568	0.0	1.02 (0.90; 1.16)	0.773	6.3	1.03 (0.95; 1.12)	0.442	0.0
Entire pregnancy															
FAs, µg/day															
400–999	Ref.			Ref.			Ref.			Ref.			Ref.		
<400	−9.52 (−38.06; 19.02)	0.513	52.4	−0.41 (−1.98; 1.16)	0.607	22.0	−0.03 (−0.20; 0.15)	0.775	86.9	0.95 (0.77; 1.17)	0.624	70.0	1.05 (0.85; 1.29)	0.666	83.9
≥1000	−10.94 (−30.28; 8.41)	0.268	1.0	−0.07 (−1.83; 1.69)	0.934	41.1	−0.01 (−0.17; 0.14)	0.894	76.9	0.98 (0.80; 1.19)	0.838	56.8	1.02 (0.83; 1.26)	0.826	79.1

First period, periconceptional period of pregnancy; second period, from the fourth to the seventh month of pregnancy; FAs, folic acid supplements; INMA, Infancia y Medio Ambiente; HRT, hit reaction time (ms); HRT(SE), hit reaction time standard error (ms); omissions, omission errors (n); commission, commission errors (n); β, beta coefficient from multiple robust linear regression; 95% CI, 95% confidence interval; I2, index to quantify the degree of heterogeneity in a meta-analysis; IRR, incidence rate ratio from negative binomial regression. ^1^ Cohort-specific models were combined using meta-analysis. All models were adjusted by energy intake (in kilocalories), dietary folate intake per 100 µg/day increase, social class (I + II (high), III or IV + V (low)), educational level (primary or less, secondary or university), parity (0 or ≥1), overall tobacco exposition during the periconceptional period (no or yes), mother’s age (in years), mother’s BMI (continuous), father’s BMI (continuous), child’s sex and child’s age at K-CPT examination (in years). We used the results from the fixed-effects meta-analysis model when *I*^2^ was less than 50% and from the random-effects meta-analysis model when *I*^2^ was greater than 50%.

**Table 3:** Fully-adjusted combined association between FAs use during pregnancy and attentional function outcomes in children aged 4–5 years of the INMA cohort study according to sex; Spain, 2003–2008 (*n* boys = 664; *n* girls = 665).

	HRT			HRT(SE)			Detectability			Omissions			Commissions		
	β ^1^ (95% CI)	*p*	*I* ^2^	β ^1^ (95% CI)	*p*	*I* ^2^	β^1^ (95% CI)	*p*	*I* ^2^	IRR ^1^ (95% CI)	*p*	*I* ^2^	IRR ^1^ (95% CI)	*p*	*I* ^2^
**BOYS**															
First period															
FAs, µg/day															
400–999	Ref.			Ref.			Ref.			Ref.			Ref.		
<400	34.36 (10.01; 58.71)	0.006	0.0	2.02 (−0.56; 4.59)	0.124	0.0	0.05 (−0.03; 0.14)	0.202	16.5	1.22 (1.01; 1.47)	0.036	8.6	0.92 (0.83; 1.03)	0.153	0.0
≥1000	33.18 (6.10; 60.25)	0.016	0.0	3.31 (0.53; 6.09)	0.019	3.7	0.04 (−0.05; 0.13)	0.395	44.8	1.25 (0.91; 1.72)	0.169	60.9	0.97 (0.80; 1.18)	0.752	57.4
Second period															
FAs, µg/day															
400–999	Ref.			Ref.			Ref.			Ref.			Ref.		
<400	−1.96 (−22.06; 18.13)	0.848	0.0	−2.15 (−4.47; 0.17)	0.069	0.0	0.06 (−0.10; 0.22)	0.490	65.3	0.92 (0.78; 1.09)	0.346	0.0	1.00 (0.91; 1.09)	0.919	37.1
≥1000	−6.03 (−57.93; 45.87)	0.820	54.6	1.01 (−1.76; 3.79)	0.474	15.2	−0.07 (−0.15; 0.02)	0.121	9.2	0.96 (0.80; 1.16)	0.687	0.0	1.10 (0.99; 1.23)	0.072	49.4
Entire pregnancy															
FAs, µg/day															
400–999	Ref.			Ref.			Ref.			Ref.			Ref.		
<400	−19.19 (−56.07; 17.69)	0.308	53.2	−0.72 (−2.99; 1.56)	0.537	0.0	−0.06 (−0.20; 0.09)	0.450	61.4	0.95 (0.70; 1.27)	0.713	71.7	1.07 (0.92; 1.25)	0.405	58.2
≥1000	−21.48 (−47.27; 4.30)	0.102	0.0	−0.11 (−2.68; 2.46)	0.935	47.9	−0.05 (−0.19; 0.10)	0.521	51.6	1.00 (0.73; 1.37)	1.000	68.0	1.05 (0.84; 1.30)	0.686	71.4
**GIRLS**															
First period															
FAs, µg/day															
400–999	Ref.			Ref.			Ref.			Ref.			Ref.		
<400	6.20 (−20.88; 25.53)	0.654	0.0	0.70 (−1.82; 3.22)	0.586	0.0	0.04 (−0.17; 0.26)	0.688	77.8	1.11 (0.94; 1.30)	0.227	0.0	0.98 (0.75; 1.27)	0.855	76.0
≥1000	−4.77 (−35.07; 25.53)	0.758	0.0	0.38 (−2.44; 3.20)	0.791	0.0	0.00 (−0.15; 0.15)	0.998	50.3	1.10 (0.92; 1.31)	0.302	0.0	0.99 (0.87; 1.13)	0.847	39.6
Second period															
FAs, µg/day															
400–999	Ref.			Ref.			Ref.			Ref.			Ref.		
<400	13.04 (−9.90; 35.98)	0.265	0.0	−0.65 (−2.52; 1.21)	0.493	20.8	0.04 (−0.04; 0.12)	0.357	0.0	0.86 (0.74; 1.00)	0.045	0.9	0.95 (0.86; 1.06)	0.358	5.3
≥1000	−4.36 (−34.59; 25.86)	0.777	0.0	−0.65 (−3.46; 2.17)	0.654	29.3	0.03 (−0.07; 0.13)	0.534	0.0	1.02 (0.86; 1.22)	0.792	0.0	0.97 (0.86; 1.11)	0.669	0.0
Entire pregnancy															
FAs, µg/day															
400–999	Ref.			Ref.			Ref.			Ref.			Ref.		
<400	10.59 (−15.21; 36.40)	0.421	0.1	0.28 (−2.14; 2.69)	0.822	0.0	0.03 (−0.19; 0.25)	0.774	80.0	1.00 (0.85; 1.18)	0.976	0.0	1.03 (0.87; 1.10)	0.837	79.5
≥1000	−3.07 (−32.22; 26.08)	0.837	22.4	−0.18 (−2.83; 2.48)	0.896	0.0	0.03 (−0.13; 0.20)	0.710	52.6	0.97 (0.81; 1.17)	0.772	0.0	1.00 (0.80; 1.27)	0.937	53.8

First period, periconceptional period of pregnancy; second period, from the fourth to the seventh month of pregnancy; FAs, folic acid supplements; INMA, Infancia y Medio Ambiente; HRT, hit reaction time (ms); HRT(SE), hit reaction time standard error (ms); omissions, omission errors (n); commission, commission errors (n); β, beta coefficient from multiple robust linear regression; 95% CI, 95% confidence interval; *I*^2^, index to quantify the degree of heterogeneity in a meta-analysis; IRR, incidence-rate ratio from negative binomial regression. ^1^ Cohort-specific models were combined using meta-analysis. All models were adjusted by energy intake (in kilocalories), dietary folate intake per 100 µg/day increase, social class (I + II (high), III or IV + V (low)), educational level (primary or less, secondary or university), parity (0 or ≥1), overall tobacco exposition during the periconceptional period (no or yes), mother’s age (in years), mother’s BMI (continuous), father’s BMI (continuous), and child’s age at K-CPT examination (in years). We used the results from the fixed-effects meta-analysis model when *I*^2^ was less than 50% and from the random-effects meta-analysis model when *I*^2^ was greater than 50%.

## Data Availability

The data presented in this study are available on request from the corresponding author. The data are not publicly available due to confidentiality and ethical reasons.

## Supplementary Materials

The following are available online at https://www.mdpi.com/2072-6643/13/2/327/s1, Table S1: K-CPT outcomes description.
