# Supplementary material for: The Use of Lower or Higher Than Recommended Doses of Folic Acid Supplements during Pregnancy Is Associated with Child Attentional Dysfunction at 4–5 Years of Age in the INMA Project
Source: Nutrients. 2021 Jan 23;13(2):327. doi: 10.3390/nu13020327 (PMC7912326; doi:10.3390/nu13020327)
Supplement: Supplementary file 1 [file nutrients-13-00327-s001.pdf]

Table 1. K-CPT outcomes description.

	Definition	*Type of variable*	*Expressed in*
**Omission errors**	The failure to respond to targets (everything except the soccer ball picture)	Discrete	Number of times (n)
**Commission errors**	Responses given to non-targets (the soccer ball picture)	Discrete	Number of times (n)
**Hit reaction time [HRT]**	The average speed of correct responses for the entire test.	Continuous	Milliseconds (ms)
**Standard error of the hit reaction time [HRT(SE)]**	A measure of response speed consistency.	Continuous	Milliseconds (ms)
**Detectability or attentiveness [d']**	A measure of the ability to distinguish between targets (everything except the soccer ball picture) and non-targets (the soccer ball picture).	Continuous	No units

Own elaboration from MHS K-CPT test items and normative data.
